# Analysis of Circulating Catestatin in Early Pregnancy: A Preliminary Investigation

**DOI:** 10.3390/biomedicines12112626

**Published:** 2024-11-16

**Authors:** Zdenka Sunjic Lovric, Jasminka Resic Karara, Bianka Mimica, Marko Kumric, Daniela Supe-Domic, Roko Santic, Josko Bozic

**Affiliations:** 1 Department of Gynecology and Obstetrics, University Hospital of Split, Spinciceva 1, 21000 Split, Croatia; zsunjic@kbsplit.hr (Z.S.L.); jreskara@kbsplit.hr (J.R.K.); bmimica@kbsplit.hr (B.M.); 2 Department of Health Studies, University of Split, Rudera Boskovica 35, 21000 Split, Croatia; dsupe@kbsplit.hr; 3 Department of Pathophysiology, University of Split School of Medicine, Soltanska 2A, 21000 Split, Croatia; marko.kumric@mefst.hr (M.K.); roko.santic@mefst.hr (R.S.); 4 Laboratory for Cardiometabolic Research, University of Split School of Medicine, Soltanska 2A, 21000 Split, Croatia; 5 Department of Medical Laboratory Diagnostics, University Hospital of Split, Spinciceva 1, 21000 Split, Croatia

**Keywords:** pregnancy, cardiovascular system, catestatin, preeclampsia

## Abstract

**Background:** During pregnancy, significant cardiovascular changes occur to accommodate fetal growth, and catestatin may play a role in these changes. Evidence suggests that catestatin, a pleiotropic sympathoinhibitory peptide, is involved in multiple cardiovascular pathologies, including hypertensive disorders. The objective of this study was to compare serum catestatin levels between first-trimester pregnant women and non-pregnant women, aiming to investigate catestatin’s role in blood pressure regulation during early pregnancy. **Methods:** This cross-sectional study included 72 first-trimester pregnant women and 57 age-matched non-pregnant controls, all without known cardiovascular or metabolic disorders. **Results:** Serum catestatin concentrations were significantly higher in pregnant women compared to controls (12.4 (9.9–21.2) ng/mL vs. 7.1 (4.5–10.9) ng/mL, *p* < 0.001). However, there was no significant difference in serum catestatin levels between those with a normal and abnormal uterine artery pulsatility index (17.8 (8.3-22.3) ng/mL vs. 12.5 (9.9–22.4) ng/mL, *p* = 0.962). Similarly, catestatin concentrations did not significantly differ between primiparous and multiparous women (14.0 (11.5–22.4) ng/mL vs. 10.7 (8.8–19.0) ng/mL). A positive correlation was observed between systolic blood pressure and serum catestatin levels in the control group (r = 0.335, *p* = 0.011) but not in pregnant women. **Conclusions:** Research on catestatin in pregnancy is still in its early stages, necessitating further studies to fully elucidate its roles and potential therapeutic applications.

## 1. Introduction

Pregnancy entails extensive physiological changes to accommodate fetal growth and development, particularly within the cardiovascular system. Key adjustments include elevated cardiac output, increased intravascular volume, and peripheral vasodilation [1]. Notably, systemic hemodynamic changes occur early in pregnancy, beginning as early as five weeks of gestation, even before placental development is complete. Additionally, alterations in maternal vascular resistance are observed before fertilization during the second phase of the menstrual cycle as a preparatory response to potential pregnancy. Cardiovascular homeostasis during this period is primarily regulated by the sympathetic nervous system and elevated estrogen levels. Therefore, an increase in sympathetic nerve activity (SNA) can be detected very early in a normotensive, physiological pregnancy [2,3]. However, there remains a significant gap in evidence-based knowledge regarding blood pressure regulation during pregnancy, especially concerning the role of the sympathetic nervous system in modulating peripheral vascular smooth muscle.

Catestatin, a product of chromogranin A, is a 21-amino acid peptide implicated in the regulation of the cardiovascular, immune, and autonomic nervous systems [4,5]. Catestatin reduces excessive sympathetic nervous system activity by inhibiting catecholamine release through the antagonism of neuronal nicotinic cholinergic receptors (nAChRs) [6,7]. However, catestatin is pleiotropic in nature, and it has also been shown to induce vasodilation by facilitating histamine release from mast cells, synergizing with adrenergic receptors to promote nitric oxide (NO) synthesis, and acting on endothelin receptors [8]. According to Fung et al., women have higher catestatin levels and more pronounced vasodilatory response to catestatin infusion when compared to men, which is an effect that seems to be mediated by estrogen [9,10].

While multiple studies have investigated the association between catestatin and hypertension, as well as preeclampsia, few have examined catestatin’s role in physiological pregnancy. Therefore, this study aims to elucidate catestatin’s potential contribution to blood pressure regulation during pregnancy by comparing serum catestatin levels in first-trimester pregnant women with those in non-pregnant controls. Additionally, this study seeks to determine whether serum catestatin levels are correlated with any clinical and laboratory characteristics of pregnant women.

## 2. Materials and Methods

### 2.1. Study Design and Ethical Considerations

This cross-sectional study was conducted at the University Hospital of Split, Croatia, between September 2022 and September 2023. The study was approved by the Ethics Committee of the University of Split, School of Medicine (Class: 500-03/22-01/120, Number: 2181-147/01/06/M.S.-22-03) and was conducted in accordance with the ethical principles of the Helsinki Declaration of 2013. Written informed consent was obtained from the pregnant women who voluntarily participated in the study after being provided with detailed information about the study’s aims and procedures.

### 2.2. Subjects and Inclusion/Exclusion Criteria

In the present study, we enrolled 72 pregnant women attending first-trimester combined screening at the Department of Gynaecology and Obstetrics, University Hospital of Split, with 57 age-matched, non-pregnant healthy women as controls. The inclusion criteria were as follows: pregnant women between 18 and 40 years old attending first-trimester combined screening, a single pregnancy, gestational age from 11 to 13+6/7 weeks and both primiparous and multiparous women. The exclusion criteria included habitual abortion, known hypertensive disorders, pregestational diabetes, oocyte donation, fetal congenital malformations, and multiple pregnancies.

### 2.3. Clinical and Laboratory Evaluations

While obtaining the mini anomaly scan during 11 to 13+6/7 weeks of gestation, the following data were collected: gravidity, parity, gestational age, height, body mass, body mass index (BMI), medical history, arterial pressure, uterine artery pulsatility index (PI), crown-rump length, and biparietal diameter. Uterine artery abnormalities were identified based on Doppler ultrasound measurements (Voluson E10), with values above the 95th percentile considered indicative of increased resistance to blood flow [11]. Subsequently, the women were referred to the Department of Medical Laboratory Diagnostics at the University Hospital of Split for biochemical analysis. The control group underwent comprehensive clinical examination and anthropometric measurements. Blood samples were collected for laboratory analysis, mirroring the procedures performed on the pregnant women, except for pregnancy-specific biochemical analyses (e.g., ß-hCG).

An altitude meter (Seca, Birmingham, UK) was used to measure height, and body mass was measured using the bioelectrical impedance scale Tanita DC-360 S (Tanita, Tokyo, Japan). The calculation of BMI was obtained according to body mass (kg) and the squared value of height (m^2^). Blood pressure was measured using the standard clinical procedure according to ESC guidelines for BP measurements. Measurement was made by a mercury sphygmomanometer with the Riva-Rocci method.

The “mini anomaly scan” calculates the risk for trisomies by taking into account maternal characteristics (primarily maternal age), biochemical measurements (free ß-hCG and PAPP-A), and ultrasound markers such as nuchal translucency, nasal bone, blood flow through the ductus venosus and possible fetal abnormalities. The gestational age was confirmed by the last menstrual period and sonographic measurement of the crown-rump length (CRL).

After an overnight fast, approximately fifteen milliliters of blood was obtained by an experienced laboratory technician. Total cholesterol, triglyceride, HDL cholesterol, LDL cholesterol, hemoglobin levels, and fasting plasma glucose were measured immediately. Serum samples were stored at −80 °C for further analysis of catestatin levels by EIA (Phoenix Pharmaceuticals Inc., Burlingame, CA, USA, Catalog #: EK-053-27CE).

### 2.4. Statistical Analysis

All statistical analyses were performed using SPSS (version 29.0, IBM, Chicago, IL, USA), and graphical representations were created with Prism 6 for Windows^®^ (version 9.4.1, GraphPad, La Jolla, CA, USA). Quantitative data were reported as the mean ± SD or median and interquartile range, as appropriate. The Shapiro–Wilk test assessed data distribution normality. Quantitative variables were compared using Student’s *t*-test or the Mann–Whitney U test, depending on data normality, while categorical data were compared using the chi-squared test and presented as n (%). Spearman’s rank correlation analysis explored correlations between catestatin and various clinical and laboratory values. Multiple linear regression was employed to determine if serum catestatin concentrations were independently associated with relevant clinical values, checking for multicollinearity using the variance inflation factor. Statistical significance was defined as *p* < 0.05 for all comparisons.

A sample size analysis was conducted prior to the study onset using data from a pilot study involving 10 pregnant women and 10 healthy controls. Serum catestatin levels, the primary outcome measure, were used to estimate the effect size. Based on these preliminary estimates, a sample size of at least 50 participants per group was calculated to achieve 90% power and a type I error rate of 0.05, allowing for the detection of a standardized difference between the groups.

## 3. Results

A total of 72 pregnant women and 57 age-matched healthy control participants were included in the present study. The comparative analysis of baseline characteristics between the group of pregnant women and the healthy control group is presented in Table 1, whereas pregnancy-related baseline characteristics are delineated in Table 2. Pregnant women had lower systolic blood pressure (*p* < 0.001), hemoglobin levels (*p* < 0.001), LDL (*p =* 0.018), and fasting plasma glucose (*p* < 0.001) in comparison to the healthy control group. Conversely, pregnant women had higher HDL (*p* < 0.001) and triglyceride levels (*p* = 0.014).

**Figure 1:**
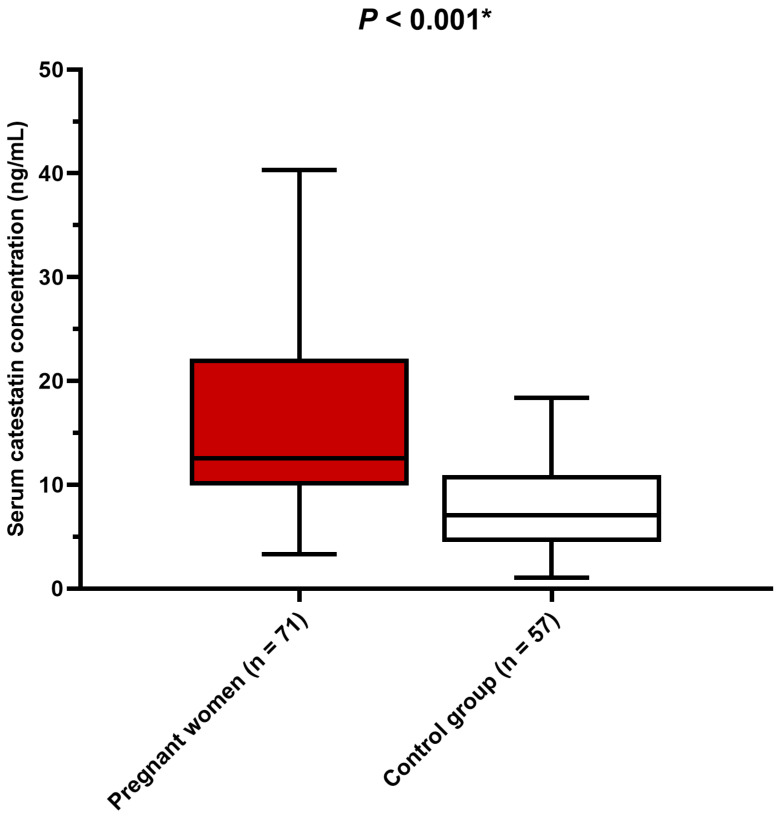
Comparison of serum catestatin concentrations between pregnant women and non-gravid control group. Data are presented as median and interquartile range. * Mann–Whitney U test.

Serum catestatin concentrations were significantly higher in pregnant women in comparison to the control group of women (*p* < 0.001) (Figure 1). Accordingly, pregnancy status was associated with serum catestatin concentrations independent of age, BMI, smoking status, and blood pressure.

**Figure 2:**
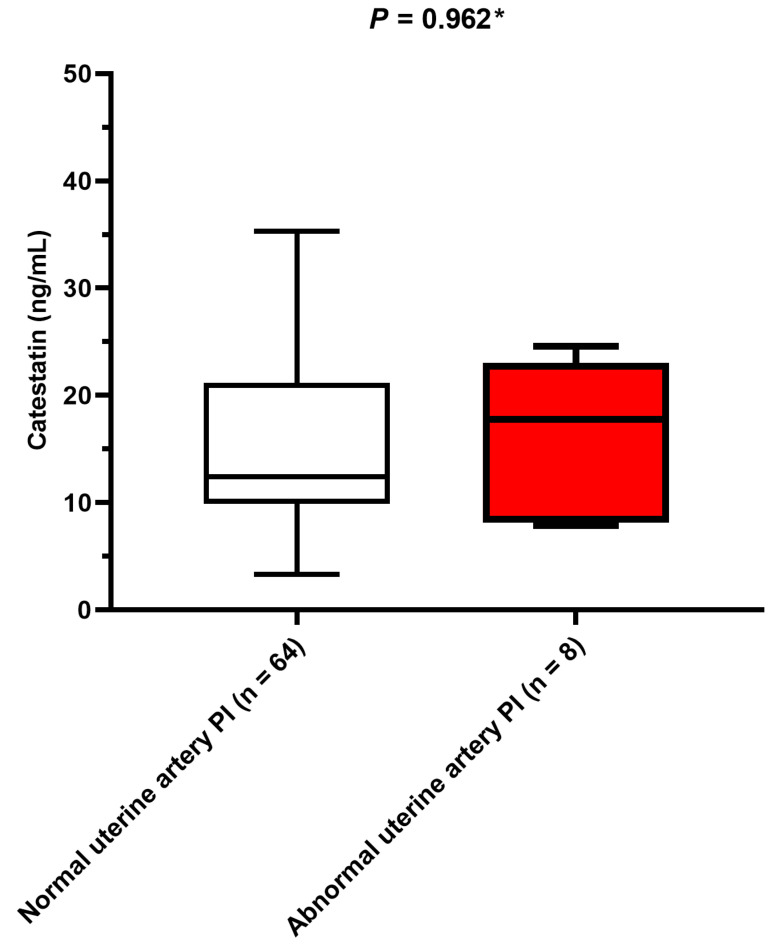
Comparison of serum catestatin concentrations between pregnant women with abnormal and normal uterine artery PI. PI: pulsatility index. Data are presented as median and interquartile range. * Mann–Whitney U test.

Serum catestatin levels did not differ between patients with normal and abnormal uterine artery PI (*p* = 0.962) (Figure 2).

**Figure 3:**
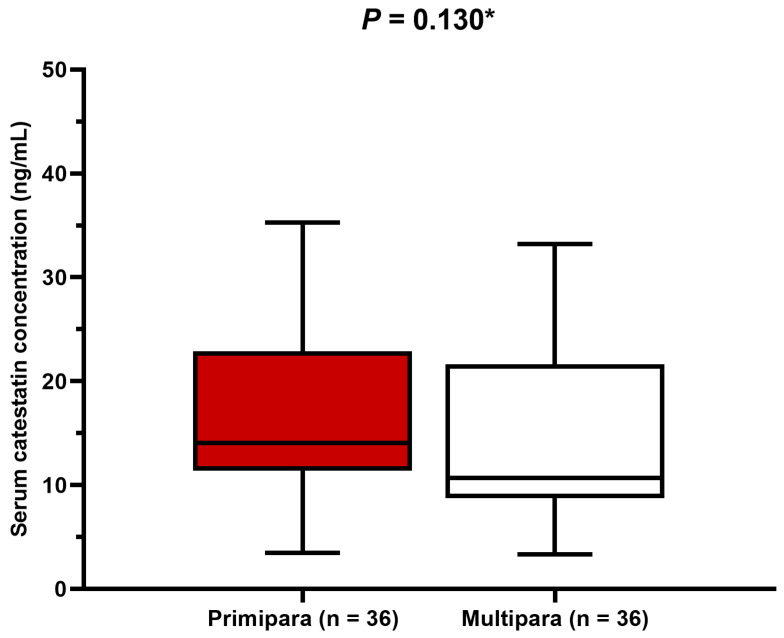
Comparison of serum catestatin concentrations between primigravidas and multiparous women. Data are presented as median and interquartile range. * Mann–Whitney U test.

There was no significant difference between primiparas and multiparous women in terms of serum catestatin levels (*p* = 0.130) (Figure 3).

While no significant correlations were found between catestatin serum concentrations and various clinical and laboratory parameters in pregnant women, systolic blood pressure was found to positively correlate with catestatin serum levels in the control group (r = 0.335, *p* = 0.011) (Table 3).

## 4. Discussion

Our study revealed significantly elevated serum catestatin levels in pregnant women compared to the control group, even after adjustment for age, BMI, blood pressure, and smoking status. On the other hand, a significant difference in catestatin levels was not found between primigravidas and multiparous women, nor did they depend on the presence of abnormal uterine artery PI. Finally, a positive correlation was observed between systolic blood pressure and catestatin serum levels in the control group but not in pregnant women. These findings contribute to the growing body of evidence suggesting that catestatin, derived from CgA, may have important implications in pregnancy and vascular health.

As catestatin has been recognized as an important factor in SNA and blood pressure regulation in non-pregnant adults, multiple studies have investigated the role of CgA and its derived peptides, including catestatin, in pregnancy and its hypertensive complications [12,13,14]. It is widely accepted that circulating CgA levels increase with the course of gestation [15]. Bralewska et al. examined the relationship between chromogranin A and preeclampsia by comparing the placentas of preeclamptic mothers with those of healthy controls. Their findings revealed a significantly higher expression of the chromogranin A gene in the placentas of preeclamptic pregnancies [16]. Additionally, protein analysis showed significantly lower catestatin levels in samples from women with preeclampsia despite no significant difference in CgA levels between the groups. These results were supported by Palmrich et al., who also found reduced serum catestatin concentrations in preeclamptic women compared to healthy pregnant women [17]. In contrast, a study by Tüten et al. reported elevated serum catestatin levels in women with both mild and severe preeclampsia compared to normotensive controls [18]. Özalp et al. did not find a significant difference in serum catestatin between preeclamptic and healthy gravid women, although the authors chose to report it as a difference without statistical significance [19]. These differences might stem from the fact that preeclamptic women constitute a very heterogeneous population, and the inclusion criteria for the above-mentioned studies are not identical.

The presence of a positive correlation between catestatin and blood pressure in the control group but not in pregnant women is worth exploring. Our previous work consistently demonstrated a positive association between ambulatory blood pressure and catestatin levels, as catestatin levels even accompanied a reduction in blood pressure following intervention [12,20]. However, considering the pleiotropic nature of catestatin, the lack of a similar correlation in pregnant women suggests that additional factors influence catestatin physiology during pregnancy. This pattern has also been observed in other conditions, such as atrial fibrillation [21]. A potential explanation lies in the adaptations of SNS operating in both normotensive and hypertensive pregnancies. Specifically, the aforementioned plasma volume expansion and systemic vasodilation observed in normal pregnancy are compensated by an increase in sympathetic activity [2]. Muscle sympathetic nerve activity (MSNA) increases during normotensive pregnancy, beginning in the first trimester and further increasing in the third trimester, suggesting that significant sympathetic activation is a characteristic of early pregnancy in humans, occurring within the first few weeks of conception, despite reduced diastolic pressure and total peripheral resistance [3]. However, despite the rise in MSNA, healthy pregnancies exhibit reduced neurovascular transduction, and such a reduction is believed to stem from increased NO levels and receptor density for vasodilators, alterations in α1- and β1-adrenergic receptor sensitivity and/or density, and decreased concentrations of signaling molecules within the neurovascular junction [22,23,24]. Numerous research groups have explored the mechanisms behind the increase in SNA during pregnancy [3,25,26,27]. Hormonal changes, particularly those involving estradiol and progesterone, are believed to contribute to the systemic vasodilation observed in pregnancy [28]. Estradiol is hypothesized to cause general vasodilation, influencing peripheral vascular resistance and arterial stiffness, which reach their lowest points in the second trimester of a normotensive pregnancy. Although estradiol has been shown to increase renal SNA in male rats, a similar effect has not been conclusively demonstrated in human pregnancy [29]. However, a longitudinal study of two pregnant women suggested a positive correlation between estradiol and MSNA [30,31]. Although vasodilatory properties of catestatin are shown in both animal and human models, whether this is a significant contributor to vasodilation in pregnancy remains elusive. The finding that there is no difference between nulliparous and multiparous women suggests that the catestatin “spike” during pregnancy remains consistent and does not diminish or escalate with subsequent pregnancies.

The increase in SNA also seems to be involved in pregnancy complications. Schobel et al. explored whether increased sympathetic vasoconstrictor activity contributes to the development of preeclampsia by measuring postganglionic SNA in the blood vessels of skeletal muscle. The study included nine women with preeclampsia, eight normotensive pregnant women, six normotensive non-pregnant women, and seven hypertensive non-pregnant women. Women with preeclampsia showed a significantly higher rate of sympathetic-nerve activity: over three times higher than normotensive pregnant women and more than twice as high as hypertensive non-pregnant women [25]. Most subsequent investigations have confirmed elevated baseline sympathetic activity in preeclampsia [25,26,27,28,29,30,31,32,33,34,35]. Contrary to these findings, a study by Reyes et al. found no difference in MSNA between women with and without preeclampsia [36]. However, in women with preeclampsia, elevated basal noradrenaline concentrations and a right-shifted baroreflex curve have been observed. In addition, a recent meta-analysis demonstrated that, in contrast to uncomplicated pregnancies, individuals with obesity, obstructive sleep apnea, and gestational hypertension exhibit elevated sympathetic activity, while those with gestational diabetes mellitus or preeclampsia do not [37]. These findings suggest that sympathetic dysfunction, indicated by increased noradrenaline levels, may play a role in the pathophysiology of preeclampsia. However, they challenge the notion that women with preeclampsia consistently exhibit sympathetic neural hyperactivity and imply that preeclampsia and gestational hypertension do not share entirely concordant pathophysiology. Having this in mind, we aimed to elucidate if patients with abnormal uterine PI have a more pronounced increase in serum catestatin. We found no difference in catestatin levels between patients with normal and abnormal uterine artery PI, casting doubt on the role of catestatin in preeclampsia. Overall, at present, it is not possible to accurately determine whether catestatin has different effects in gravid females compared to non-gravid females, especially considering its pleiotropic nature [38]. Mechanistically, the effect of catestatin most relevant for cardiovascular adaptation in pregnancy may be vasodilation. Additionally, we sought to determine if catestatin levels are associated with indicators of pathological pregnancies, such as elevated blood pressure and the uterine artery pulsatility index, given our previous findings regarding its role in hypertension and vascular health. Based on our results, it seems that catestatin is not associated with such indices, although the limitations of the present study must be acknowledged. To further clarify catestatin’s role in pregnancy, we are currently conducting a prospective study measuring catestatin at multiple time points throughout pregnancy. This study aims to compare the dynamics between healthy pregnancies and those affected by preeclampsia and to determine if catestatin levels could serve as a predictive marker for preeclampsia.

The primary limitation of this study is its cross-sectional design, which limits our ability to draw causal conclusions. Additionally, the non-constant secretion of catestatin further impedes the establishment of causality, given that measurements were conducted at a single time point. The single-center design and limited number of participants also hinder the generalization of the present findings.

In summary, our study identified significantly elevated serum catestatin levels in pregnant women compared to the controls, even after adjusting for possible confounders and regardless of multiparity. Despite catestatin’s established role in regulating SNA and blood pressure in non-pregnant adults, our findings indicate that its relationship with blood pressure in pregnant women is influenced by additional factors. Although our study did not find differences in catestatin levels between patients with normal and abnormal uterine artery PI, which challenges the role of catestatin in preeclampsia, the complex interplay between catestatin, sympathetic activity, and vascular adaptation in pregnancy warrants more comprehensive studies to elucidate these mechanisms.

## Figures and Tables

**Table 1:** Baseline characteristics of the study population.

Characteristic	Non-Pregnant Women (n = 57)	Pregnant Women (n = 72)	p *
Age, years	31 (27–39)	31 (29–34)	0.747
Body mass index, kg/m^2^	24.4 (22.3–26.5)	23.3 (21.5–26.1)	0.191
Smoker, n (%)	6 (10.5)	24 (31.2)	0.005
Office blood pressure			
SBP, mmHg	120 (114–126)	110 (105–120)	<0.001
DBP, mmHg	73 (68–89)	70 (69–80)	0.610
Hemoglobin, g/L	129.5 ± 7.3	135.7 ± 11.4	<0.001
Fasting blood glucose, mmol/L	4.96 ± 0.48	4.52 ± 0.47	<0.001
Cholesterol, mmol/L	4.6 (4.2–5.5)	5.1 (4.3–5.9)	0.094
LDL cholesterol, mmol/L	2.8 (2.3–3.4)	2.6 (2.1–3.2)	0.018
HDL cholesterol, mmol/L	1.4 (1.2–1.7)	1.9 (1.5–2.2)	<0.001
Triglycerides, mmol/L	1.22 ± 0.63	1.54 ± 0.47	0.014

Abbreviations: DBP: diastolic blood pressure; HDL: high-density lipoprotein; LDL: low-density lipoprotein; SBP: systolic blood pressure. * Student’s *t*-test or Mann–Whitney test, or chi square test, as appropriate. Data are presented as mean ± SD, median (IQR) or n (%), as appropriate.

**Table 2:** Pregnancy-related baseline characteristics.

Characteristic	Pregnant Women (n = 72)
Gestation, weeks *	12 (11–14)
Gravidity, n (%)	
Primipara	36 (50)
Second	19 (26.4)
Third or more	17 (23.6)
History of miscarriage, n (%)	9 (12.5)
IVF, n (%)	3 (4.2)
Mean uterine artery PI (Left)	1.6 ± 0.6
Mean uterine artery PI (Right)	1.7 ± 0.6
Abnormal uterine artery PI, n (%)	8 (11.1)
CRL, mm	62.3 ± 7.6
NB, n (%)	66 (91.7)
NT, mm	1.1 (1.0–1.7)
BPD, mm	21.0 (19.9–23.4)
BetaHCG [MoM]	0.98 (0.62–1.39)
PAPP-a [MoM]	1.4 (0.9–2.0)
NT [MoM]	0.72 (0.64–0.93)

Abbreviations: IVF: in vitro fertilization; PI: pulsatility index; CRL: crown-rump length; NB: nasal bone; NT: nuchal translucency; BPD: biparietal diameter; BetaHCG: beta human chorionic gonadotropin; PAPP-a: pregnancy-associated plasma protein A; MoM: multiples of the median. * Median (min–max). Data are presented as mean ± SD, median (IQR), or n (%), as appropriate.

**Table 3:** Correlation between catestatin serum concentrations and various clinical and laboratory parameters.

Parameter	r—Correlation Coefficient *	*p*
Age	0.153	0.200
Left uterine artery PI	0.019	0.879
Right uterine artery PI	−0.036	0.769
Fasting plasma glucose	−0.127	0.288
LDL cholesterol	0.107	0.426
SBP (gravid women)	−0.008	0.489
SBP (control group)	0.335	**0.011**
BMI	−0.080	0.504
Beta-HCG (MoM)	0.062	0.606
PAPP-A (MoM)	−0.028	0.819
NT (MoM)	−0.065	0.594

Abbreviations: LDL cholesterol: low-density lipoprotein cholesterol; SBP: systolic blood pressure; BMI: body mass index; Beta-HCG: Beta-Human Chorionic Gonadotropin; PAPP-A: pregnancy-associated plasma protein A; NT: nuchal translucency. MoM: multiples of the median. Values in bold indicate statistical significance. Correlation analysis for age, BMI, and LDL pertains to gravid patients. * Spearman’s rank correlation.

## Data Availability

The data presented in this study are available upon request to the corresponding author. The data are not publicly available because some of the data sets will be used for further research.
